# Whole-Genome Sequence of Cyanobacterium *Oxynema* sp. AP17, Isolated from an Indian Mangrove Forest

**DOI:** 10.1128/MRA.00808-20

**Published:** 2020-10-29

**Authors:** Shayontani Basu, Veerabadhran Maruthanayagam, Arnab Pramanik, Joydeep Mukherjee

**Affiliations:** a School of Environmental Studies, Jadavpur University, Kolkata, India; b Department of Biochemistry, University of Calcutta, Kolkata, India; University of Maryland School of Medicine

## Abstract

*Oxynema* sp. AP17 is the second distinct species of the *Oxynema* genus, and it differs from the first (*Oxynema thaianum*) in 16S-23S internal transcribed spacer (ITS) sequence, morphology, and optimal salinity. The genome size is 6.37 Mbp; 4,801 protein-coding genes, 74 tRNAs, and 5 each of the 16S, 23S, and 5S rRNAs constitute the genome.

## ANNOUNCEMENT

Six cyanobacteria of the Indian Sundarbans have been assigned to the *Lyngbya-Phormidium-Plectonema* (LPP) group B and one each to the *Oscillatoria* and *Synechocystis* genera based on morphological features (1). Two strains (AP17 and AP24) of the eight cyanobacteria previously isolated by us (2) were established as Oxynema aestuarii species of the recently described genus *Oxynema* (3) by our workgroup (4) based on molecular, ecological, and morphological differences. Isolation involved aseptic collection of cyanobacterial soil surface biofilm, inoculation in artificial sea nutrient (ASN-III) medium, incubation with fluorescent irradiance (50 μmol of photons·m^−2^·s^−1^) in a 12-h:12-h light:dark cycle at 25 ± 1°C, and plating of 10^4^-fold serially diluted homogenized biomass obtained after 40 days. Individual colonies of filamentous cyanobacteria were isolated after 30 days on ASN-III plates, observed microscopically, and grown in liquid ASN-III medium supplemented with 1 ml of cycloheximide (from 50 mg liter^−1^ of stock) and 20 ml of triple-antibiotic solution (containing penicillin G, chloramphenicol, and streptomycin sulfate in a 10:5:10 ratio) per 1,000 ml of liquid medium to prevent culture contamination. This cyanobacterial cell suspension was subsequently grown in antibiotic-free ASN-III medium for 30 days, and culture purity was ascertained by the absence of microbial growth in tryptone-yeast extract-glucose (TYG) broth (1, 2). Here, we report the whole-genome sequence of cyanobacterium *Oxynema* sp. AP17, isolated from the Indian Sundarbans.

*Oxynema* sp. AP17 monoculture was regularly subcultured on solid and liquid ASN-III media every 15 to 21 days since its isolation and incubated under the light and temperature conditions mentioned previously (5). Freshly passaged cells maintained in solid and liquid media for 21 days were used for DNA extraction.

Genomic DNA extracted with the Qiagen QIAamp DNA minikit (catalog number 51306; Venlo, Netherlands) was fragmented, damaged ends were repaired, and adaptors were ligated to the fragments. Library preparation was done following the standard PacBio 20-kb protocol, and fragments having a size of >10 kb were selected with BluePippin (Sage Science, MA). PacBio Sequel sequencing employing P6-C4 chemistry yielded 590,344 reads and 1,280,423 subreads with average lengths of 6,000 bp and 6,830 bp, respectively. Subreads were assembled using the BBTools suite (http://sourceforge.net/projects/bbmap/). Default parameters were used except where otherwise noted. The reads were *de novo* assembled using Canu v2.0 (6). Assembly review and refinement, potential artifact removal, read QC, adaptor trimming, error correction, and overlap filtering (using parameter --bestn –) were performed using the SMRT Link Microbial Assembly application (SMRT Link v9.0) (https://www.pacb.com/wp-content/uploads/SMRT_Tools_Reference_Guide_v90.pdf, https://www.pacb.com/wp-content/uploads/Application-Brief-Microbial-whole-genome-sequencing-Best-Practices.pdf, and https://github.com/PacificBiosciences) and Circlator (https://sanger-pathogens.github.io/circlator/) for contig circularization and rotation of the genome around the *dnaA* gene. The final polished data were obtained as a single circular 6,357,750-bp assembly with a G+C content of 51.5%.

The genome sequence was annotated using the NCBI Prokaryotic Genome Annotation Pipeline (PGAP version 4.11) (7). The genome consisted of 4,801 protein-coding genes, 74 tRNAs, and 5 each of the 16S, 23S, and 5S rRNAs.

### Data availability.

The genome sequence of *Oxynema* sp. AP17 is available in NCBI (accession number CP051167). The raw sequence is available from SRA number SRX8430131 under the BioProject number PRJNA614629 and BioSample number SAMN14410527.
